# A Weibull distribution-based method for estimating seed longevity in *Solanum rostratum*

**DOI:** 10.3389/fpls.2026.1700839

**Published:** 2026-02-26

**Authors:** Zhili Yuan, Weidong Fu, Zhen Song, Zhonghui Wang, Chengyu Sun, Yue Zhang, Guoliang Zhang

**Affiliations:** Institute of Environment and Sustainable Development in Agriculture, Chinese Academy of Agricultural Sciences, Beijing, China

**Keywords:** invasive weed management, longevity modeling, persistent seed bank, seed-bank regulation, viability decline

## Abstract

**Introduction:**

Seed longevity is a key determinant of population persistence, spread, and outbreak potential in annual invasive plant species. Understanding longevity of invasive seed bank is crucial for determining colonization timing and assessing invasion potential, thereby supporting sustainable weed management strategies. While soil seed bank fluctuations have become a focus in invasion biology area, efficient and accurate methods for evaluating seed bank longevity in annual invasive plants remain scarce so far. In this study, we focus on a representative annual globally malignant invasive plant *Solanum rostratum*, investigating seed longevity by accelerated aging test (60°C and 85% relative humidity) across multiple regions and collection years.

**Methods:**

We used a three-parameter Weibull distribution model to characterize seed aging and applied it to assess *S. rostratum* seed bank longevity in both grassland and abandoned farmland habitats.

**Results:**

The results showed that *S. rostratum* seeds lost viability rapidly within 3 d under accelerated aging condition. Seeds from different regions in the same year exhibited similar aging patterns, while interannual variation led to significantly divergent aging curves. Based on polynomial regression of viability data and germination tests, the upper limit of seed longevity under natural field conditions was estimated to be approximately 8–9.79 years.

**Discussion:**

This study demonstrates that combining accelerated aging assays with the three-parameter Weibull distribution provides an effective approach for assessing seed longevity and soil seed bank persistence. The method offers a practical, efficient, and reproducible framework for estimating seed bank persistence in annual invasive plants. Our findings highlight the critical role of persistent seed banks in facilitating the invasion success of *S. rostratum*, thereby offering a robust analytical basis for evaluating invasion risks. Moreover, the modeling framework developed here can be extended to other annual plant species for seed viability assessment, providing valuable theoretical support for the development of ecologically sustainable weed management strategies.

## 1 Introduction

Persistent soil seed banks serve as a fundamental driver of invasion success in annual invasive plant species. Defined as reservoirs of viable seeds and vegetative propagules capable of regenerating plant communities (Abella et al., 2013), seed banks play a vital role in maintaining future weed populations (Bossuyt and Honnay, 2008). Previous studies demonstrates that many annual invasive species produce seeds capable of surviving extended periods in soil (Passos et al., 2017), maintaining viability under adverse conditions while rapidly germinating when conditions improve (Moravcová et al., 2022). This delayed germination strategy preserves population genetic diversity and enhances adaptability to environmental variability (Gioria et al., 2019).

The soil seed banks of annual invasive plants sustain invasive population persistence while severely reducing native seed bank species richness and seed density (Traba et al., 2004; Gioria et al., 2014). With prolonged invasion, ecological restoration resistance increases, as accumulated invasive seeds create major barriers to vegetation recovery (Schuster et al., 2018). Preventing further seed bank replenishment is therefore critical. Systematic investigations into invasive seed bank characteristics are essential for assessing invasion risks and re-invasion potential (Gioria et al., 2019), while also guiding the development of region-specific, cost-effective management strategies (Schwartz-Lazaro and Copes, 2019).

The lifespan or longevity of a seed is the time period over which it can remain viable (Nadarajan et al., 2023), while seed persistence is the survival of seeds in the environment once they have reached maturity (Long et al., 2015). Seed longevity, although not equivalent to seed persistence, is a critical component for assessing the persistence potential of this species in natural environments. However, current approaches to assessing seed viability in annual invasive plants are inadequate. Most studies focus on seed bank composition, density, and ecological roles (Vandvik et al., 2016; DeMalach et al., 2021; Yang et al., 2021), while research on longevity remains limited. Traditional methods can only interpret seed viability and face major constraints. Germination assays, though widely used, cannot determine seed age or initial quantity and are biased in species with complex dormancy such as *S. rostratum* (Dairel and Fidelis, 2020). Seed burial experiments, while partly simulating natural dynamics, are labor-intensive, artificial, and impractical for deeply dormant species (Mickelson and Stougaard, 2003; Saatkamp et al., 2009). These shortcomings highlight the need for more efficient and reliable methods to evaluate invasive seed bank longevity and persistence.

Studies demonstrate that high-temperature/humidity aging treatments effectively simulate natural seed deterioration through shared physiological mechanisms (Walters, 1998; Walters et al., 2010), providing a practical approach to rapidly assess seed viability in annual invasive species. The Accelerated aging (AA) test – a standard method for evaluating seed vigor – expose seeds to elevated temperatures (typically 40 ~ 50°C) and approximately 100% relative humidity (RH), inducing rapid degradation (Tekrony, 1993; Woltz and Tekrony, 2001). High-vigor seeds exhibit slower viability decline under these conditions while maintaining higher germination rates. Unlike crop seeds, certain invasive species exhibit significantly slower aging rates at 40 ~ 50 °C, requiring temperatures above 50 °C to effectively accelerate deterioration (Fenollosa et al., 2020). Monitoring viability changes under such conditions allows researchers to infer seed storage potential, longevity, and physiological quality. Recent applications of the AA test in invasive plant control studies (Company et al., 2019) highlight its utility in ecological management. The three-parameter Weibull distribution has proven particularly effective for modeling seed longevity. For instance, it successfully characterized viability in *Carpobrotus edulis*’ seeds (Fenollosa et al., 2020). This distribution’s flexibility and superior model fit (Watt et al., 2010) make it ideal for describing asymmetric viability decline patterns. Its probability density function (PDF) is defined as:


$$\begin{array}{c} f(x;\lambda ,k,c)=e^{-{\left(\frac{x-c}{\lambda }\right)}^{k}} \end{array}$$

In this distribution, *x* ≥ *c*, and the density function ( x; λ, k, c) equals zero when *x* < *c*. The primary distinction between the three parameter Weibull distribution and the standard two-parameter version lies in the inclusion of a location parameter *c*, which enhances model flexibility and allows for better fitting of skewed data sets. This enables precise quantification of seed storage potential and longevity through viability analysis, with particular effectiveness in modeling asymmetric viability decline patterns. Although originally developed for reliability analysis in engineering fields – such as memory devices, fatigue resistance in mechanical systems, and aerospace structures – the three-parameter Weibull distribution has also been shown to effectively describe seed germination rates and germination speed (Bridges et al., 1989). In seed science, its simplicity and adaptability (Watt et al., 2010) have made it an important mathematical tool for modeling seed viability and longevity. Compared with other nonlinear models, such as Gompertz and Logistic functions, the three-parameter Weibull distribution demonstrates superior fitting accuracy and lower sensitivity to initial seed viability values, resulting in more stable and reliable estimates (Brown and Mayerf, 1988). Therefore, it is well-suited for accurately estimating the seed longevity of annual invasive plant species.

Traditional methods for assessing seed viability historically relied on germination test. However, Germination tests are time-consuming, strongly influenced by environmental conditions, and unable to distinguish dormant from dead seeds, which may lead to underestimation of seed viability. To enhance the precision of viability evaluation in accelerated aging tests, the tetrazolium (TZ) test has emerged as a complementary approach. Widely adopted for seed viability testing due to its rapidity, sensitivity, and broad applicability (França-Neto and Krzyzanowski, 2022; Qiao et al., 2023; Setty et al., 2023), this method was specifically optimized in this study for the annual invasive species *S. rostratum*. Building on protocols established for its close relative species *Solanum melongena* (Yu et al., 2015), the refined tetrazolium staining protocol enabled precise tracking of seed vigor during aging.

*Solanum rostratum*, a globally malignant invasive annual weed native to Mexico and the central United States (Whalen, 1979), has colonized regions across North America, Europe, Africa, Asia, and Oceania, threatening ecosystem stability and agroecological security (Rushing et al., 1985; Eminniyaz et al., 2013). This species exhibits exceptional reproductive capacity, with individual plants producing 1,600 ~ 43,800 seeds (Whalen, 1979; Wei et al., 2009), and readily forms persistent soil seed banks. Approximately 55% of seeds germinate in the first post-sowing spring, while the remainder germinate in subsequent growing seasons (Shalimu et al., 2012), around 20% entering long-term dormancy to sustain seed bank longevity. The seeds (2.2~2.8 mm diameter) possess a dense, honeycomb-patterned coat (Lin, 2007) that enhances permeability and mechanical resistance (Yang et al., 2019), coupled with notable stress tolerance (Yu et al., 2021). These traits facilitate a dual physical-physiological dormancy mechanism (Wei et al., 2010), enabling the establishment of large, persistent seed banks – a critical driver of its ongoing global invasion success.

Seed longevity serves as a critical indicator for assessing the persistence, spread, and outbreak potential of annual invasive plants within specific habitats. Despite its ecological significance, systematic evaluation of seed longevity in such species remains lacking. *S. rostratum* was selected as a model organism for this study due to its representative seed traits, ease of sampling, and dual theoretical-practical relevance. This research aims to: (1) develop an accelerated aging system for *S. rostratum* seeds under high-temperature/humidity conditions to rapidly assess viability, (2) analyze viability patterns across seed collection years and geographic origins to identify key longevity determinants, (3) model aging dynamics using the three-parameter Weibull distribution, calculating L_50_ (time to 50% viability loss) and estimating natural longevity via polynomial regression, (4) assess invasion plants’ seed persistence by analyzing soil seed bank L_50_ values across habitats. By elucidating seed longevity mechanisms and spatiotemporal viability trends, this study provides a scientific framework for long-term invasive weed management, technical support for tracing invasion histories, evidence-based strategies for evaluating control measures, and region-specific management protocols for invasive species.

## 2 Materials and methods

### 2.1 Seed viability assessment

#### 2.1.1 Optimization of tetrazolium staining duration for *S. rostratum* seed viability assessment

All experiments were conducted from 1 August to 28 September 2023. Seeds used in this study were collected from different regions and years and stored under dry conditions at 4°C. Detailed information on seed origin, collection date, storage duration, and experimental use of each seed lot is provided in **Table 1**. Mature *S. rostratum* seeds from the 2022 seed lot (**Table 1**) were used for optimization of tetrazolium (TZ) staining time. Fifty seeds were placed into each of nine 5 mL centrifuge tubes. The 1% 2, 3, 5 - triphenyltetrazolium chloride (TTC) solution was added to each tube, followed by incubation in a 40°C water bath. At time points of 12, 24, and 36 h, three replicates per time point were removed from the bath. After discarding the TTC solution via pipetting, seeds were rinsed 1~2 times with distilled water, longitudinally sectioned with a scalpel and forceps, and microscopically examined for staining patterns. The influence of staining duration on coloration quality was assessed, and representative staining images were compiled to determine the optimal staining time for use in subsequent accelerated aging experiments.

**Table 1:** Collection history, storage conditions, and experimental use of *Solanum rostratum* seed lots.

Seed lot	Region	Coordinates	Collection date	Storage conditions	Storage duration	Experimental use
2008	Shuangta District, Chaoyang City, Liaoning Province	120˚28’47” E, 41˚36’36” N	2008.09	Dry storage at 4°C	179 months	AA (storage-year comparison)
2015	Zhenlai County, Baicheng City, Jilin Province	122°51’0” E, 45°34’12” N	2015.09	Dry storage at 4°C	95 months	AA (storage-year comparison)
2019	Tuquan county, Xing ‘an League, Inner Mongolia Autonomous Region	121˚35’24” E, 45˚22’48” N	2019.09	Dry storage at 4°C	47 months	AA (storage-year comparison)
2020	Beipiao City, Chaoyang City, Liaoning Province	120˚55’34” E, 41˚30’25” N	2020.09	Dry storage at 4°C	35 months	AA (storage-year comparison)
2021/TZ	Tianzhen county, Datong city, Shanxi Province	114°4′48″ E, 40°25′12″ N	2021.09	Dry storage at 4°C	23 months	AA (storage-year comparison); AA (regional comparison)
BY	Urat Front Banner, Bayannur City, Inner Mongolia Autonomous Region	108°39′0″ E, 40°43′11″ N	2021.09	Dry storage at 4°C	23 months	AA (regional comparison)
CJ	Changji city, Changji Hui Autonomous Prefecture, Xinjiang Uygur Autonomous Region	87°18′0″ E, 44°1′12″ N	2021.09	Dry storage at 4°C	23 months	AA (regional comparison)
GY	Guyang County, Baotou City, Inner Mongolia Autonomous Region	110°3′24″ E, 41°1′47″ N	2021.09	Dry storage at 4°C	23 months	AA (regional comparison)
HT	Huimin District, Hohhot city, Inner Mongolia Autonomous Region	111°37′26″ E, 40°48′29″ N	2021.09	Dry storage at 4°C	23 months	AA (regional comparison)
TY	Tongyu county, Baicheng city, Jilin province	123°4′48″ E, 44°49′12″ N	2021.09	Dry storage at 4°C	23 months	AA (regional comparison)
2022	Huaian County, Zhangjiakou City, Hebei Province	114°23′8″ E, 40°40′27″ N	2022.09	Dry storage at 4°C	11 months	TZ staining time optimization; AA (temperature optimization); AA (storage-year comparison)
2023	Yanqing District, Beijing City	115°51′22″ E, 40°23′38″ N	2023.09	Dry storage at 4°C	0 months	AA (storage-year comparison)

Storage duration refers to the period from seed collection to the start of experiments in August 2023.

#### 2.1.2 Quantitative method for viability assessment using tetrazolium staining

According to the Tetrazolium Testing Handbook (Association of Official Seed Analysts (AOSA), 2000), the criteria for determining seed viability are as follows:

Viable seeds (normal staining): The embryo is uniformly and fully stained without rupture; slight damage to the radicle is acceptable; the radicle may appear slightly darker; small and lightly stained or darkly stained areas may occur at the periphery of the hypocotyl or cotyledons; more than half of the cotyledons remain attached to the hypocotyl and are evenly stained.

Non-viable seeds (abnormal or no staining): The embryo shows no staining; the tissue appears loose and lacks firmness; the radicle is unstained, degenerated, or ruptured above the tip of the central vascular tissue; the inner surface of the cotyledons shows unstained or water-soaked dark areas; less than half of the cotyledon area remains functional and attached to the hypocotyl; or the hypocotyl is damaged.

**Figure 1:**
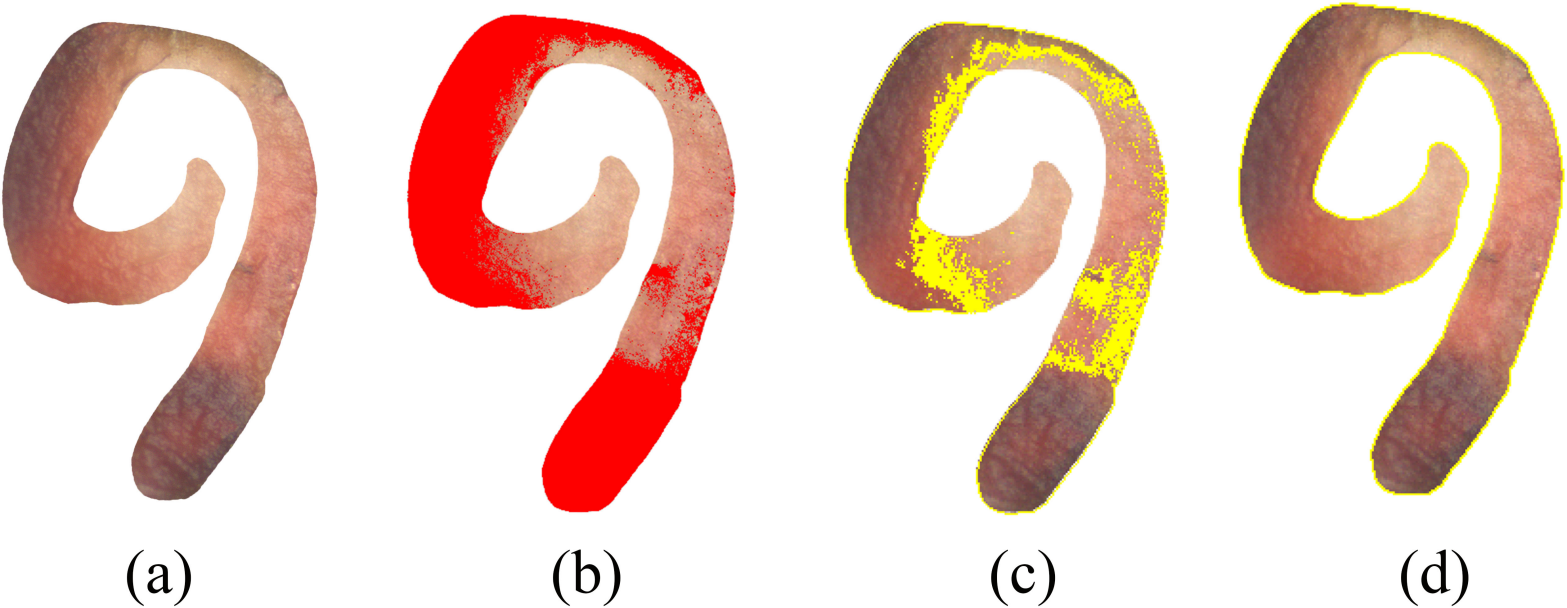
Procedures for accurately measuring the stained area of seed cross-sections. **(a)** stained section; **(b)** selecting red-stained regions in Fiji; **(c)** calculating the red-stained area; **(d)** calculating the total area.

Besides, considering visual assessment can be subject to observer bias, seed embryo images in this study were quantified using FIJI software. The procedure was as follows (**Figure 1**):

1) File → Open: Import the tetrazolium-stained embryo image.
2) Image → Adjust → Color Threshold: Adjust Hue, Saturation, and Brightness until all red-stained regions are selected (highlighted in red).
3) Under Threshold Color → Select, choose Analyze → Measure to obtain the stained embryo area.
4) Image → Adjust → Threshold: Adjust the threshold so that the entire embryo cross-section is selected (shown in red).
5) Select Analyze → Measure to obtain the total embryo area.
6) Calculate staining percentage as:
7) Stained area (%) = (Stained embryo area/Total embryo area) × 100.
8) Seeds with < 50% stained embryo area were classified as non-viable, and those ≥ 50% were classified as viable.

#### 2.1.3 Determination of optimal accelerated aging temperature

To shorten the experimental duration and improve efficiency, an evaluation was conducted to determine the optimal temperature for accelerated aging of *S. rostratum* seeds. Seeds from the 2022 seed lot (**Table 1**) were subjected to aging treatments at four temperatures (45°C, 50°C, 55°C, and 60°C) under a constant relative humidity of 85%. For each temperature treatment, 18 replicates were prepared, each consisting of 50 seeds evenly placed in mesh bags and laid flat in an aging incubator (LH-1509). For the 45°C, 50°C, and 55°C treatments, samples were taken every 3 d; for the 60°C treatment, sampling was performed daily. At each sampling point, 3 replicates were assessed using the tetrazolium test method to evaluate seed viability.

### 2.2 Accelerated aging experiment across populations and collection years

To evaluate both geographical variation and collection-year effects on the seed longevity of *S. rostratum*, two accelerated aging (AA) experiments were conducted. One experiment compared seed lots collected from different geographical regions in 2021, while the other compared seed lots collected in different years (**Table 1**). Prior to accelerated aging, all seed lots were pre-imbibed in distilled water for 24 h at room temperature to ensure full hydration before exposure to high temperature and humidity conditions.

The optimized protocol (Section 2.1.1) was applied across all seed lots. Seeds were placed in mesh bags, with three biological replicates per population or collection year (50 seeds per replicate), and incubated at 60°C and 85% RH. Sampling was conducted at 0, 24, 48, 72, and 96 h. Seed viability was assessed using the TTC staining protocol optimized in Section 2.1.1, and viability values were recorded for subsequent modeling of aging.

### 2.3 Application of accelerated aging tests in soil seed bank research

To evaluate the applicability of the accelerated aging test conditions and the three-parameter Weibull distribution method established in this study under field-relevant conditions, soil seed bank samples of *S. rostratum* were collected from two distinct habitats:

Grassland habitat: Ke’erqin Grassland in Taobei District, Baicheng City, Jilin Province (45°34′12″N, 122°51′0″E).

Abandoned farmland habitat: Huai’an County, Zhangjiakou City, Hebei Province (40°40′27″N, 114°23′8″E).

Soil cores were collected from 0–10 cm and 10–20 cm depths. After cleaning, drying, and sieving (10- and 14-mesh), visually identifiable black kidney-shaped *S. rostratum* seeds were manually selected. A total of 400 seeds per habitat were obtained.

These soil-derived seeds were subjected to the same accelerated aging protocol described in Section 2.1. Three biological replicates (50 seeds each) were sampled from 0 to 96 h at 24-hour intervals, and viability was assessed using the TTC staining method. These data were used to evaluate model performance when applied to naturally aged seeds from soil seed banks.

### 2.4 Seed aging modeling framework

In this study, L_50_ is defined as the time required for seed viability to decline to 50% under the accelerated ageing conditions used here, and is used as a quantitative metric for comparing relative longevity among *S. rostratum* populations within this experimental framework. The seed viability were modeled with a three-parameter Weibull distribution, whose probability density function is:


$$\begin{array}{c} f(x;\lambda ,k,c)=e^{-{\left(\frac{x-c}{\lambda }\right)}^{k}} \end{array}$$

Where x≥c, and when x<c, the density function f(x; λ, k, c) is equal to 0. Here, *λ* represents the scale parameter of the three-parameter Weibull distribution, *k* represents the shape parameter of the three-parameter Weibull distribution, and *c* is the threshold parameter of the three-parameter Weibull distribution. *x* represents the accelerated aging time, and f(x) represents the seed viability at the corresponding time.

### 2.5 Longevity estimation

For each population or collection year, seed longevity was quantified using the estimated L_50_ values derived from the three-parameter Weibull model. To infer the potential upper limit of seed persistence under natural conditions, the L_50_ values obtained from different collection years were fitted with a nonlinear regression model. The resulting fitted curve was used to extrapolate longevity trends and estimate the maximum potential seed lifespan of *S. rostratum* in field environments.

### 2.6 Validation of the seed longevity model using germination tests

To validate the predictions of the accelerated aging test model, seeds collected in 2008, 2015, and 2023 were subjected to gibberellic acid (GA_3_) treatment following the method of Wei et al. (2010). For each collection year, six replicates of 30 seeds were prepared. Seeds were soaked in a GA_3_ solution (8 g·L^-^¹) for 24 h under dark conditions and then sown in pots filled with a standard potting substrate. The pots were maintained at room temperature (25°C) under a 12 h light/12 h dark photoperiod, and watered every three days to maintain adequate soil moisture. Germination was initiated three days after sowing and recorded daily until no further germination occurred. A seed was considered germinated when the radicle emerged ≥ 2 mm beyond the seed coat—a criterion consistent with ISTA’s radicle emergence test for fast viability assessment (Ermis et al., 2022).

### 2.7 Data processing and statistical analysis

All raw data—including viability measurements from condition screening, accelerated aging experiments, soil seed bank assays, and germination validation tests, were compiled and organized i in Microsoft Excel 2023. Statistical analyses were conducted using SPSS 27.0 and MATLAB R2024a, and Origin 2023.

For each staining category (red, mottled, and white), differences among staining durations (12/24/36 h) were tested using one-way ANOVA. When significant effects were detected, Tukey’s HSD *post hoc* test was applied for multiple comparisons. Statistical significance was set at p < 0.05. Different lowercase letters indicate significant differences among treatments, whereas identical letters indicate no significant difference.

Seed viability data were fitted using a three-parameter Weibull model, implemented in MATLAB R2024a using the Curve Fitting Toolbox (fit, fittype, and fitoptions). Model selection was conducted by comparing Akaike’s Information Criterion (AIC) and the Bayesian Information Criterion (BIC) among competing non-linear models, including a two-parameter Weibull model, a GLM-based logistic regression model (Starbuck, 2023), and a Gompertz model. AIC/BIC calculations and model comparisons were performed in MATLAB using the Statistics and Machine Learning Toolbox, with information criteria derived based on residual sums of squares (RSS). Models with lower AIC/BIC values were considered to provide a better balance between goodness-of-fit and parsimony. From each fitted Weibull curve, the time required for viability to decline to 50% (L_50_) was estimated and used as an index of seed longevity. L_50_ values were then calculated from accelerated ageing curves for seeds collected in different years and regressed against chronological seed age. Because this L_50_–age regression was used to infer an upper bound of longevity based on laboratory-derived L_50_ values, rather than to represent a direct physiological or ecological process, model selection prioritized goodness-of-fit; therefore, a second-order polynomial regression was adopted to describe the relationship between L_50_ and seed age. This regression and model evaluation were also conducted in MATLAB (Curve Fitting Toolbox/Statistics and Machine Learning Toolbox).

To evaluate differences in seed longevity among populations and among collection years, L_50_ values derived from individual experimental replicates were used as the response variable in subsequent statistical analyses. One-way analysis of variance (ANOVA) was performed with population (for regional comparisons) or collection year (for storage-year comparisons) as the independent factor. Prior to ANOVA, the normality of L_50_ values was assessed, and no data transformation was required. Because statistical comparisons were conducted on continuous L_50_ estimates rather than on raw proportional viability or germination data, binomial distribution assumptions were not applicable. Statistical significance was determined at p < 0.05.

Figures presenting experimental results, including condition screening and accelerated aging assays, were generated using Origin 2023, whereas Weibull model simulations and polynomial regression were produced using MATLAB R2024a.

## 3 Results

### 3.1 Optimal conditions for rapid viability testing of *S. rostratum* seeds

#### 3.1.1 Optimal staining duration for evaluating *S. rostratum* seed viability using tetrazolium test

**Figure 2:**
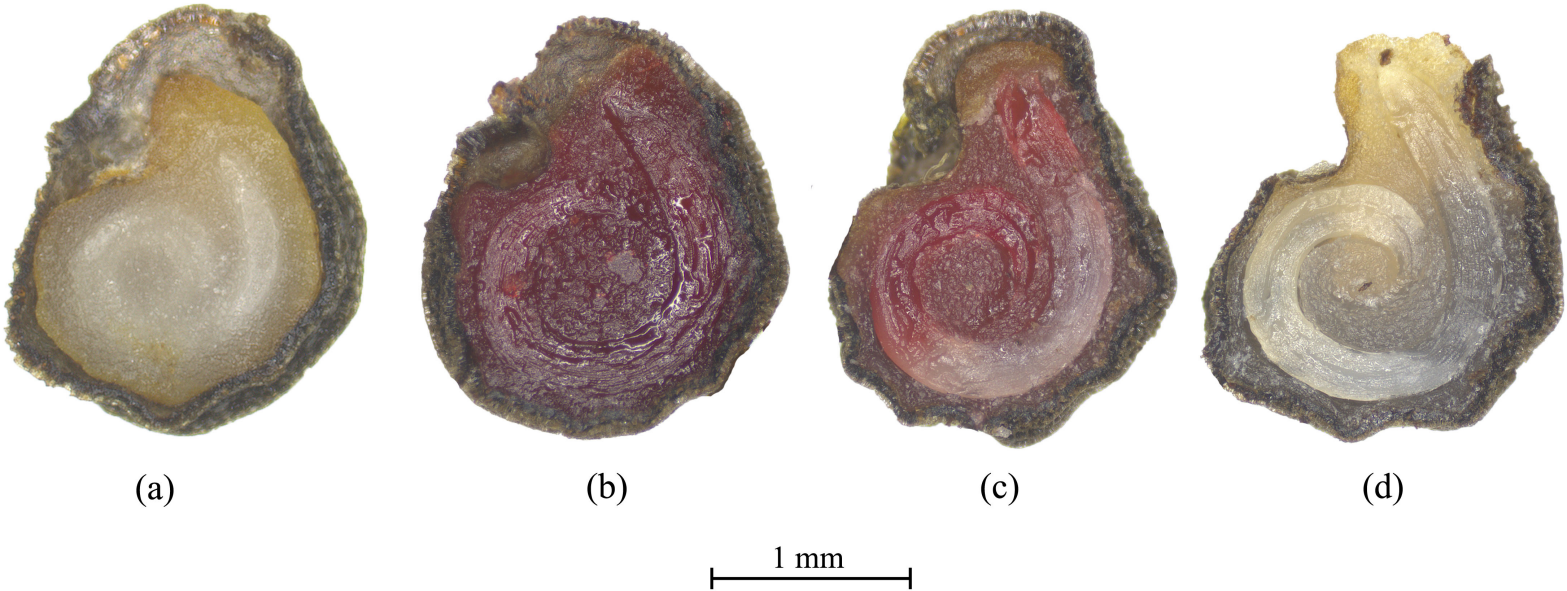
Diagnostic correlation between tetrazolium staining patterns and physiological viability in *Solanum rostratum* seeds. **(a)** Negative control: Cross-sectional view of an unstained seed under standardized observation conditions (seeds collected in 2023). **(b)** Viable seed: The embryo and endosperm exhibit uniform bright red staining, confirming active dehydrogenase-mediated reduction of 2,3,5-triphenyltetrazolium chloride **(TTC)** (seeds collected in 2023). **(c)**: Non-viable (Type I): The embryonic axis shows partial carmine staining with necrotic mottling, indicating localized cellular degradation (seeds collected in 2023). **(d)**: Non-viable (Type II): Embryonic tissues demonstrate complete absence of chromogenic reaction, diagnostic of irreversible metabolic arrest (seeds collected in 2008).

To establish a standardized protocol for assessing *S. rostratum* seed viability via the tetrazolium test, this study defined three distinct viability categories based on staining patterns (**Figure 2**). Viable seeds exhibited uniform bright red staining of both the embryo and endosperm with intact tissue morphology. Seeds displaying partial or unstained embryos combined with more than 50% unstained or structurally compromised storage tissues were classified as non-viable (mottled-stained). Fully unstained seeds with softened, decayed, or damaged tissues were categorized as non-viable (dead).

**Figure 3:**
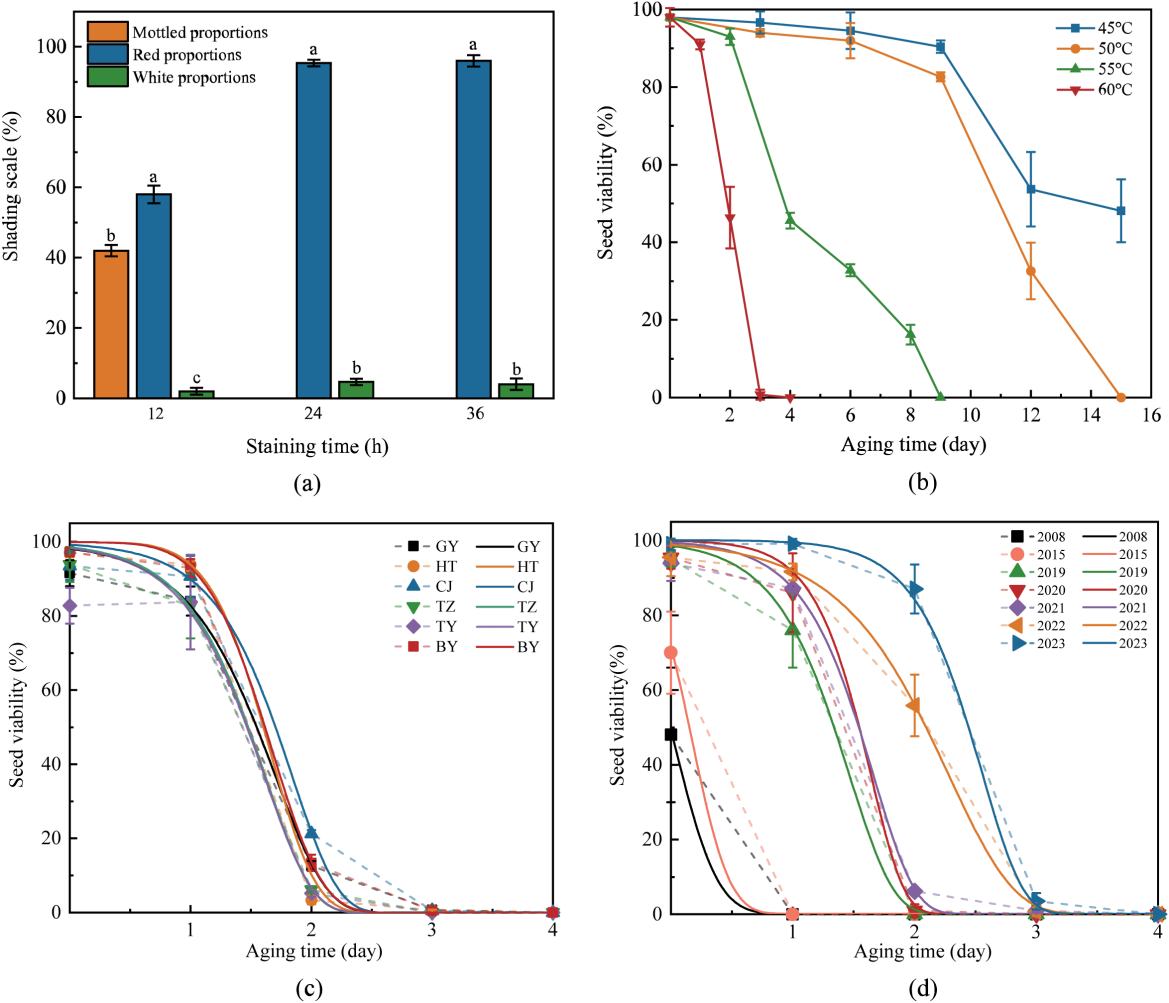
Variations of *Solanum rostratum* seed viability under accelerated aging conditions. **(a)** Temporal viability dynamics quantified by tetrazolium staining rate (%) during 0–96 h aging. **(b)** Correlation between aging duration **h**, thermal stress, and viability decay trajectories. **(c)** Regional variation in seed viability loss kinetics among six geographical populations subjected to standardized accelerated aging conditions. **(d)** Interannual variation in seed viability for seed lots collected between 2008 and 2023 under standardized accelerated aging conditions (60°C, 85% relative humidity). Lowercase letters denote statistically significant intergroup differences (P < 0.05). Geographical codes: GY: Guyang County (Inner Mongolia), HT: Hohhot City (Inner Mongolia), CJ: Changji City (Xinjiang), TZ: Tianzhen County (Shanxi), TY: Tongyu County (Jilin), BY: Bayannur City (Inner Mongolia). Symbols represent observed mean viability values (± SD), with vertical error bars indicating standard deviation, and solid lines represent fitted three-parameter Weibull model curves.

Staining duration significantly influenced diagnostic accuracy. Time-course experiments (40°C, 1% TTC) revealed suboptimal results at 12 h, with only 51.8% viable seeds, 44.8% mottled-stained, and 3.2% dead (**Figure 3a**). The high mottled-stained proportion indicated incomplete enzymatic reduction of TTC. In contrast, 24-hour and 48-hour treatments achieved 96.6% and 96.8% viable seeds, respectively, with complete elimination of mottled staining. Given negligible improvement beyond 24 h, a 24-hour staining duration at 40°C was selected as the optimal balance between efficiency and precision for subsequent experiments.

#### 3.1.2 Optimal temperature for rapid viability assessment of *S. rostratum* seeds

**Figure 3b** illustrates the viability decline curves of *S. rostratum* seeds under varying temperature treatments (all maintained at 85% relative humidity). The results demonstrate that temperature profoundly influences the aging process, modulating both the survival curve morphology and the median viability period L_50_.

At the minimal test temperature of 45°C, seeds exhibited progressive deterioration, showing approximately 40% viability reduction over 15 d. Comparative analysis revealed comparable deterioration rates between 45°C and 50°C conditions, both treatments reached 50% viability on day 9, though diverging thereafter – 45°C seeds maintained gradual decline, while 50°C specimens underwent rapid depletion to complete non-viability by day 15. Thermal acceleration became pronounced at 55°C, with viability initiating exponential decay on day 3 (L_50_ attainment: day 4; full viability loss: day 9). The most extreme aging occurred at 60°C, where viability demonstrated precipitous decline within 24 h, culminating in complete depletion by day 4.

Collectively, under sustained 85% humidity, incremental temperature elevation (from 45°C to 60°C) reduced L_50_ values from 15 d to less than 2 d, confirming thermal sensitivity as a critical driver of viability loss. To maximize efficiency in viability assessment and aging sensitivity analysis, 60°C with 85% relative humidity was selected as the optimal accelerated aging condition. This protocol enables comprehensive seed viability evaluation within one week.

#### 3.1.3 Comparison of the three-parameter Weibull distribution with other survival models

Using the viability data obtained from accelerated aging experiments across different geographical populations, we compared the fitting performance of the three-parameter Weibull distribution with that of the two-parameter Weibull, Logistic, and Gompertz survival models. Model selection metrics, including AIC and BIC, are summarized in **Table 2**. The results showed that the three-parameter Weibull model provides the best overall fit among the tested models. Therefore, the viability decline pattern of *S. rostratum* seeds is appropriately characterized by the three parameter Weibull distribution.

**Table 2:** Model comparison based on AIC and BIC.

	AIC				BIC			
Site	Three-parameter Weibull distribution	Two-parameter Weibull distribution	Logistic	Gompertz	Three-parameter Weibull distribution	Two-parameter Weibull distribution	Logistic	Gompertz
GY	-93.25	-88.63	-86.55	-86.35	-91.13	-86.51	-85.43	-85.23
HT	-84.85	-84.85	-83.97	-83.96	-82.72	-82.72	-81.85	-81.84
CJ	-93.56	-91.86	-91.80	-91.71	-91.43	-89.74	-88.68	-88.59
TZ	-89.07	-86.83	-85.57	-85.57	-86.95	-84.70	-83.44	-83.44
TY	-67.23	-65.62	-64.86	-64.82	-65.10	-63.49	-61.73	-61.70
BY	-114.30	-114.30	-113.09	-113.91	-112.18	-112.17	-110.97	-110.79

AIC and BIC are the Akaike and Bayesian information criteria, respectively, which compare model performance on the same dataset while penalizing model complexity (number of parameters). Lower AIC and BIC indicate a better fit.

#### 3.1.4 Geographic consistency in seed viability dynamics of *S. rostratum*

Under standardized accelerated aging conditions (60°C, 85% RH), interregional seeds of *S. rostratum* collected within the same year exhibited conserved viability loss patterns. As illustrated in **Figures 3c**, all populations demonstrated triphasic degradation kinetics: gradual viability reduction during the initial 24-hour phase (0 ~ 1 day) followed by precipitous decline from day 2 onward, culminating in complete viability loss by day 2 ~ 3. The consistent pattern across populations indicates that, although seed longevity is influenced by environmental conditions, it remains relatively stable across different sites. This stability may represent a distinctive trait of the invasive species *S. rostratum*, contributing to its persistence and successful colonization.

To quantitatively characterize these dynamics, viability time-series data were fitted to the three-parameter Weibull survival function. The resultant models (**Figure 3c**) demonstrated excellent goodness-of-fit, with near-identical decay parameters observed among geographically distinct populations. This convergence underscores the robustness of the aging mechanism under controlled environmental stress.

Based on the three-parameter Weibull distribution, L_50_ values (time required for viability to decline to 50%) were calculated for each of the six populations (**Figure 3c**; **Table 3**). All L_50_ values were below 2 d, indicating that *S. rostratum* seeds lose viability rapidly under high temperature and humidity. Estimated L_50_ values for the six populations were narrowly distributed between 1.46 and 1.69 d, with largely overlapping 95% credible intervals (**Table 3**). One-way ANOVA detected no significant differences in L_50_ among the six populations (p = 0.104 > 0.05, **Table 4**). These results suggest that seeds collected in the same year display relatively consistent longevity under accelerated aging conditions.

**Table 3:** Quantitative parameters of Three-parameter Weibull distribution and L_50_ values characterizing *Solanum rostratum* seed viability across geographical populations.

Population abbreviations	*λ*	*k*	*c*	L_50_ (d)(95% credible interval)
GY	139.35	329.10	-137.65	1.54 (1.39,1.55)
HT	1.97	6.11	-0.26	1.59 (1.47,1.70)
CJ	49.17	130.19	-47.33	1.69 (1.56,1.71)
TZ	62.61	163.43	-61.00	1.47 (1.31,1.48)
TY	216.71	551.84	-215.10	1.46 (1.22,1.58)
BY	1.96	5.54	-0.22	1.61 (1.56,1.64)

*λ* represents the scale parameter of the three-parameter Weibull distribution, *k* represents the shape parameter of the three-parameter Weibull distribution, and *c* represents the threshold parameter of the three-parameter Weibull distribution. Geographical codes: GY: Guyang County (Inner Mongolia), HT: Hohhot City (Inner Mongolia), CJ: Changji City (Xinjiang), TZ: Tianzhen County (Shanxi), TY: Tongyu County (Jilin), BY: Bayannur City (Inner Mongolia).

**Table 4:** One-way ANOVA of L_50_ among *Solanum rostratum* populations from different geographical regions.

Source	df	SS	MS	F	p
Between groups	5	0.128	0.026	2.358	0.104
Within groups	12	0.130	0.011	–	–
Total	17	0.258	–	–	–

One-way ANOVA was based on replicate-level L_50_ values (three replicates per population).

#### 3.1.5 Chronological aging patterns in *S. rostratum* seeds

Accelerated aging trials revealed divergent viability decay kinetics among *S. rostratum* seeds stratified by collection year (**Figure 3d**). Freshly harvested seeds and one-year-old seeds exhibited progressive deterioration commencing on day 1 of thermal-hydric stress. In stark contrast, seeds aged 2–4 years displayed catastrophic viability collapse within the first 24 h, reaching near-complete non-viability by day 2. Notably, 8–15 year-old seeds entered experiments with substantially compromised initial viability and achieved total non-viability within 24 h.

Temporal viability profiles were robustly modeled using the three-parameter Weibull survival function (**Figure 3d**, **Table 5**), demonstrating excellent goodness-of-fit. Derived L_50_ values exhibited strong negative correlation with storage duration: maximal longevity occurred in fresh seeds (L_50_ = 2.39 d), followed by 1-year-old seeds (2.06 d). Seeds stored for less than 2 years had L_50_ values greater than 2 d, while those stored for 3–4 years had L_50_ values between 1–2 d. For seeds stored for more than 5 years, L_50_ values fell below 1 day, indicating extreme sensitivity to aging. A one-way ANOVA of L_50_ values obtained from three-parameter Weibull fits to the accelerated aging data showed a highly significant effect of collection year on seed longevity (p = 0< 0.05), indicating that seeds collected in different years differed strongly in their inherent persistence (**Table 6**).

**Table 5:** Three-parameter Weibull distribution model parameters and L_50_ values characterizing *Solanum rostratum* seed viability across collection years.

Collection year	*λ*	*k*	*c*	L_50_ (d)(95% credible interval)
2008	1.03	3.56	-0.94	0 n.e.
2015	0.97	3.93	-0.75	0.10 n.e.
2019	64.12	180.11	-62.66	1.20 (-3.86,6.27)
2020	24.23	89.61	-22.59	1.46 (1.27,1.54)
2021	68.35	202.70	-66.69	1.45 (1.41,1.50)
2022	119.10	225.66	-116.82	2.06 (1.98,2.14)
2023	1.03	3.56	-0.94	2.39 (2.32,2.47)

*λ* represents the scale parameter of the three-parameter Weibull distribution, *k* represents the shape parameter of the three-parameter Weibull distribution, and *c* represents the threshold parameter of the three-parameter Weibull distribution. n.e. = not estimable. For these populations, seed viability dropped almost instantaneously under accelerated aging, resulting in insufficient information for reliable model fitting.

**Table 6:** One-way ANOVA of L_50_ among *Solanum rostratum* populations from different years.

Source	df	SS	MS	F	p
Between groups	6	14.459	2.410	410.431	0.000
Within groups	14	0.082	0.006	–	–
Total	20	14.541	–	–	–

One-way ANOVA was based on replicate-level L_50_ values (three replicates per population).

These findings establish a quantifiable inverse relationship between chronological age and aging resistance in *S. rostratum* seeds. The L_50_ metric functions as a dual indicator of both storage history and physiological competence, providing critical insights for seed bank longevity modeling and invasion timeline reconstructions.

#### 3.1.6 Modeling and validation of seed longevity in *S. rostratum*

**Figure 4:**
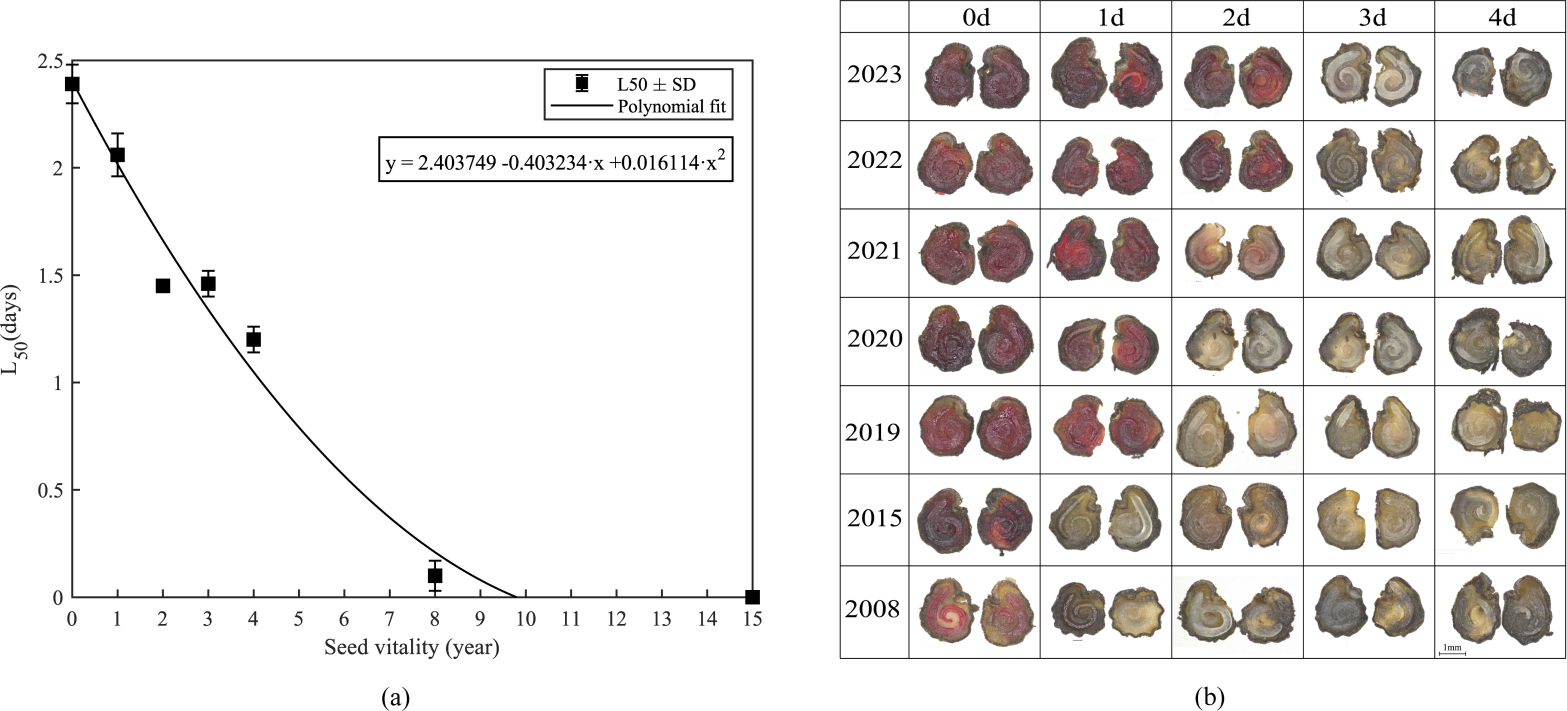
Interannual variation in seed longevity and viability of *Solanum rostratum*. **(a)** Polynomial regression modeling of median seed survival time (L_50_) in relation to chronological age for *Solanum rostratum* seed seeds. The analysis quantifies longevity trajectories across differentially aged seed populations. **(b)** Interannual viability dynamics of *Solanum rostratum* seeds under accelerated aging condition. Tetrazolium staining profiles of seeds from 2008–2023 seeds after 96-hour aging (60°C, 85% RH). Seeds with both embryo and non-embryonic tissues stained bright red are considered viable, while those with mottled or no staining in these tissues are classified as non-viable.

Polynomial regression was applied to relate L_50_ values (**Figure 4a**), calculated from accelerated aging curve models of seeds from different collection years, to their corresponding seed ages. A significant negative relationship was observed between seed age and L_50_, with a coefficient of determination close to unity (R² = 0.98036) and a minimal residual sum of squares (RSS = 0.09672), indicating a very high level of model fit (**Table 7**).

In the regression analysis, two intercepts were obtained when L_50_ was extrapolated to zero, at 9.79 and 15.23 years. Germination test results showed that seeds collected in 2015 (8-year-old seeds) exhibited a germination rate of 10%, whereas seeds collected in 2008 (15-year-old seeds) failed to germinate completely, indicating a loss of viability. Therefore, we infer that when stored in a laboratory refrigerator at 4°C, the upper limit of *S. rostratum* seed longevity is likely to be approximately 8–9.79 years.

Complementary histochemical analysis (**Figure 4b**) revealed age-dependent viability patterns. Seeds aged less than 6 years maintained stable initial viability but exhibited progressive staining attenuation during aging, while older cohorts (≥ 7 years) showed accelerated degradation kinetics. Distinct temporal gradients in tetrazolium staining intensity correlated precisely with seed age, enabling visual differentiation of senescence stages.

**Figure 5:**
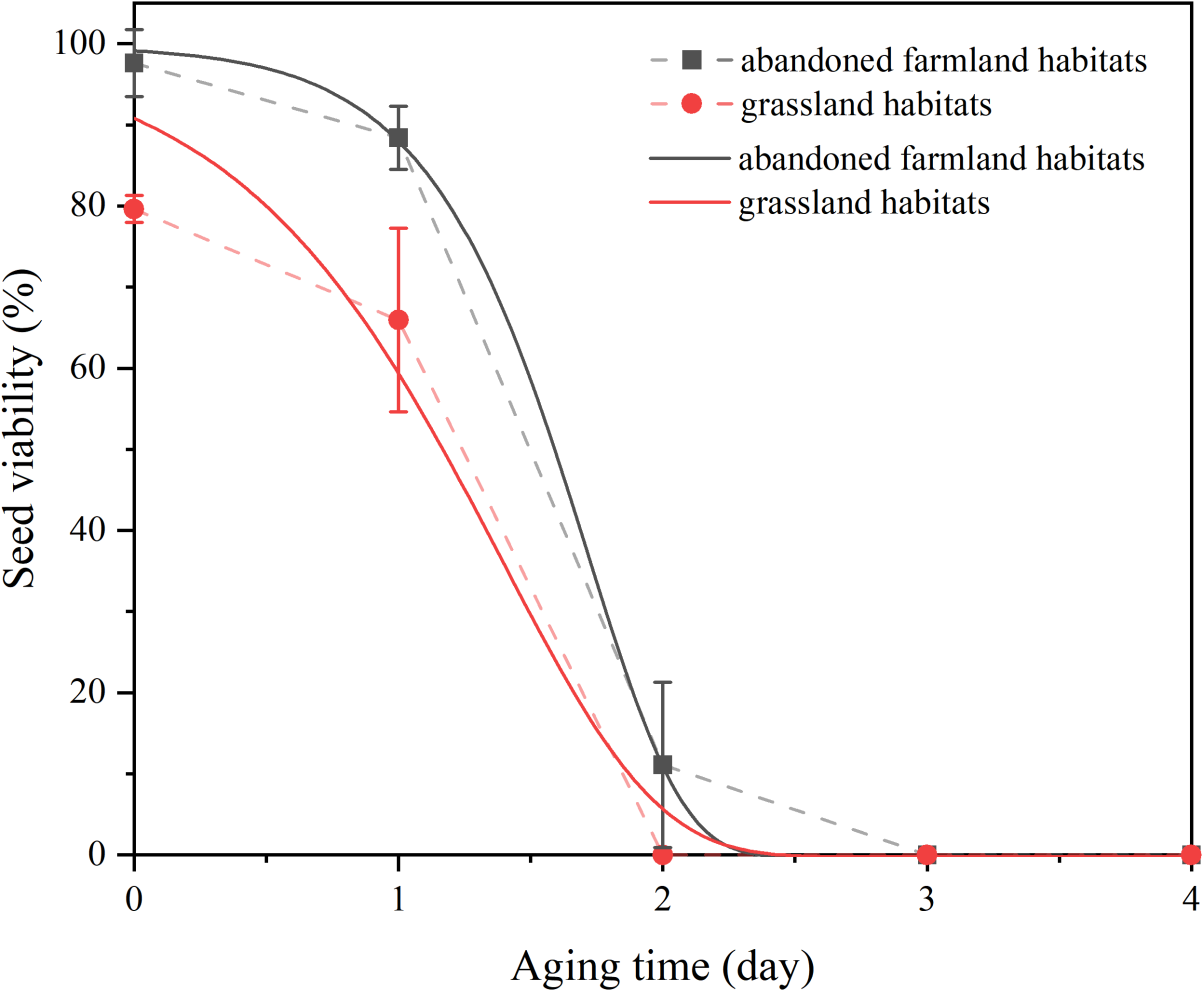
Viability of *Solanum rostratum* soil seed bank viability under accelerated aging conditions across two habitat types. Symbols represent mean observed viability values (± SD) during 0–96 h of accelerated aging under standardized conditions (60°C, 85% relative humidity). Vertical error bars indicate standard deviation, and solid lines represent fitted three-parameter Weibull model curves.

This study provides technical support for the rapid determination of *S. rostratum* seed age. In practical applications, soil samples collected from the field should be brought back to the laboratory, where the recovered seeds can be subjected to accelerated aging treatment and their viability patterns compared with the reference images in **Figure 5** to estimate their age range. By integrating viability curves with tailored control strategies, targeted management measures can be developed for different invasion stages of *S. rostratum*, thereby enhancing the effectiveness of weed control and informing ecological management decisions for invasive species. Variation in seed viability of *S. rostratum* across different habitats.

The seed viability of *S. rostratum* exhibited significant variation across habitats. As illustrated in **Figure 5**; **Tables 7**, **8**, seeds collected from abandoned farmland demonstrated an L_50_ value of 1.59 d. Polynomial fitting analysis estimated the seed bank longevity (y-value) in this habitat at 2.39 years, indicating viability comparable to seeds stored for approximately two years. In contrast, seeds from grassland habitats showed a lower L_50_ value (1.36 d) with a corresponding longevity estimate of 3.11 years, equivalent to seeds stored for around three years.

**Table 7:** Polynomial fitting equations and parameter estimates.

Parameter	Value	Standard error
Equation	$y=Intercept+B1\times x+B2\times x^{2}$	
Intercept	2.403749	± 0.112197
B1	-0.403234	± 0.046211
B2	0.016114	± 0.002934
Residual Sum of Squares (RSS)	0.096720	
Adjusted R-squared	0.98036	

The residual sum of squares (RSS) represents the sum of the squared differences between the observed and predicted values in the regression model. A smaller RSS indicates a better model fit. The coefficient of determination (R²) reflects the proportion of variance in the dependent variable explained by the model, ranging from 0 to 1, with values closer to 1 indicating a better fit.

**Table 8:** Three-parameter Weibull distribution model parameters and L_50_ values quantifying *Solanum rostratum* seed viability dynamics across different habitats.

Habitat	*λ*	*k*	*c*	L_50_ (d)(95% credible interval)
Abandoned farmland	22.46	63.61	-20.74	1.59 (1.43,1.60)
Grassland	275.88	469.10	-274.49	1.36 (-6.26, 8.50)

*V* represents viability (%), and *x* represents aging time in days. *λ* represents the scale parameter of the three-parameter Weibull distribution, *k* represents the shape parameter of the three-parameter Weibull distribution, and *c* represents the threshold parameter of the three-parameter Weibull distribution.

Although ecological differences in seed viability exist between habitats, the three-parameter Weibull distribution model developed in this study demonstrated good fit and parameter stability for both habitat types, indicating strong explanatory power. This seed bank longevity estimation model not only enables quantitative evaluation of the survival potential of invasive species seeds in specific regions but also shows good cross-regional applicability. Application experiments using soils from two ecologically distinct locations confirmed the model’s robustness under varying environmental conditions. These findings suggest that the model can be reliably used to predict the outbreak potential of invasions across multiple regions, thereby providing a scientific basis for the development of site-specific management strategies.

### 3.2 Estimation of *S. rostratum* seed longevity

As detailed in **Table 9**, the results demonstrated that seeds collected in 2008 (15 years old) had completely lost viability, with no germination observed after 30 d. Seeds collected in 2015 (8 years old) exhibited an average germination rate of 10.6% after 7 d (P < 0.05), indicating a marked decline in viability but a residual capacity for germination. In contrast, seeds collected in 2023 (1 year old) germinated rapidly, reaching a mean germination rate of 86.7% within 7 d. These findings indicate that seed viability of *S. rostratum* had significantly declined by 2015(8 years old), while complete loss of viability occurred in the 2008 (15 years old) cohort. Our results indicate that *S. rostratum* seeds can remain viable for approximately 9.79 years under 4°C dry storage. Since dry storage at 4°C provides conditions more benign than those typically experienced in natural habitats, the estimated longevity is best interpreted as the upper bound of seed persistence under field conditions.

**Table 9:** Germination rates of seeds collected in different years.

Collection year	Sample size (n)	Germination rate (%)(Mean ± SD)	Individual replicate values (%)
2008	6	0.00 ± 0.00 a	0.00, 0.00, 0.00, 0.00, 0.00, 0.00
2015	6	10.56 ± 3.93 a	10.00, 6.67, 13.33, 6.67, 16.67, 10.00
2023	6	86.67 ± 5.16 b	83.33, 93.33, 86.67, 80.00, 83.33, 93.33

Different lowercase letters within the same column indicate significant differences among groups (P < 0.05), whereas the same letters indicate no significant difference. Values are presented as mean ± SD. Statistical significance was determined using one-way analysis of variance (ANOVA) followed by Tukey’s multiple comparison test.

## 4 Discussion

### 4.1 Optimal conditions for artificial accelerated aging

The tetrazolium test, widely used for detecting infarct areas in mammalian tissues (Bederson et al., 1986), is also effective for assessing seed viability (Towill and Mazur, 1975). The staining agent, 2,3,5-triphenyl tetrazolium chloride (TTC), is a lipid-soluble, photosensitive compound. In seeds or plant tissues, viable tissues are stained varying shades of red, whereas dead or non-viable tissues remain unstained.

Studies on *Hordeum vulgare* (Lopez Del Egido et al., 2017; Ma et al., 2019) and the artificial, self-pollinated cereal crop species ×Triticosecale Wittmack (triticale) (Souza et al., 2010) demonstrate that the tetrazolium test generates viability estimates comparable to germination tests but with significantly greater efficiency. During preliminary viability testing for *S. rostratum*, both germination and tetrazolium tests were attempted. However, after 20 d of germination trials, only untreated seeds germinated, achieving a rate of merely 1%.

For annual invasive plants like *S. rostratum* – which exhibit hard seed coats and mixed dormancy – germination tests are unsuitable for rapid viability assessment in accelerated aging experiments. Consequently, the tetrazolium test, as employed in this study, offers a more efficient and reliable alternative. In preliminary trials, we found that directly immersing intact seeds in TTC solution did not produce stable or readily interpretable staining results. This is likely because the seed coat exhibits semi-permeable properties that restrict the penetration of TTC solution into internal seed tissues (Beresniewicz et al., 1995; Zhou et al., 2013). In addition, *S. rostratum* seeds possess a relatively dark seed coat (Wei et al., 2010), which makes it difficult to clearly observe internal staining patterns without cutting. Therefore, a small longitudinal incision was made to allow the TTC solution to directly contact the embryo tissue, enabling clear longitudinal observation of embryo staining while minimizing any potential impact on embryo viability. By optimizing staining conditions and protocols, the tetrazolium test effectively evaluates *S. rostratum* seed viability, providing robust data for subsequent research.

Accelerated aging tests are designed to impose extreme conditions of temperature and humidity to simulate long-term seed deterioration within a short period of time. In many crop studies, 100% relative humidity has been adopted, such as 50°C/100% RH for rice (*Oryza sativa*) (Guo et al., 2022), 42°C/100% RH for barley (*Hordeum vulgare*) (Ebone et al., 2019), and 41°C in saturated NaCl solution for chickpea (*Cicer arietinum*) (Araújo et al., 2021). However, alternative protocols using lower but still high humidity levels have also been reported. For example, tomato (*Solanum lycopersicum*), a species in the same family as *S. rostratum*, was tested at 40°C/85% RH (Vidigal et al., 2016), and for the invasive plant *Carpobrotus edulis*, 55°C/87% RH was identified as the most suitable condition (Fenollosa et al., 2020). Considering the strong stress tolerance of *S. rostratum* and the limitation of available equipment, which could reach a maximum of 85% RH, this study adopted 85% RH combined with a temperature gradient to accelerate seed deterioration. While this approach effectively captured interannual variation in seed viability, the absence of a humidity gradient remains a limitation. Future studies should therefore explore combined temperature–humidity gradients, ideally coupled with germination or seedling establishment assays, to refine predictions of seed bank persistence in invasive plants.

### 4.2 Regional variation and seed longevity

Our results indicate that longevity-related metrics derived from the L_50_ were broadly comparable among geographic populations of *S. rostratum*, suggesting limited among-population divergence within this experimental framework. In contrast, common garden experiments revealed significant differentiation (p < 0.01) among *S. rostratum* populations in multiple morphological traits, indicating strong genetic divergence (Chen et al., 2013). This discrepancy implies that seed longevity may represent a conserved and essential trait underpinning the persistence of *S. rostratum*, whereas morphological traits are more evolutionarily labile and responsive to local selective pressures. The combination of stable seed longevity and divergent morphological adaptation may together contribute to the invasion success of *S. rostratum*.

The use of accelerated aging combined with a Weibull-based modeling approach offers clear advantages for invasive species research. In a seed burial experiment, viability declined rapidly during the initial phase and then decayed much more slowly thereafter, producing an asymmetric pattern that is poorly captured by a simple exponential model but is well described by a Weibull function (Campbell et al., 2012). The viability trajectories of *S. rostratum* seeds observed in our accelerated ageing assays exhibited a similar two-phase decay; therefore, we selected the Weibull distribution as the primary model for fitting viability dynamics.

Our study demonstrates that this framework can effectively quantify viability loss patterns, providing a practical tool for predicting persistence in seed banks and supporting long-term management planning. Future studies could further strengthen the robustness of accelerated ageing assessments by conducting experiments under multiple ageing conditions, which would help evaluate the consistency of viability decline patterns and improve confidence in longevity comparisons.

From a weed management perspective, understanding that *S. rostratum* seeds exhibit consistent longevity across regions underscores the difficulty of controlling its soil seed banks through short-term interventions. Instead, integrated management strategies must account for its medium-term persistence and gradual decline, aligning with approaches used for other invasive species with resilient seed reserves.

### 4.3 Upper bound of *S. rostratum* seed longevity

Although the accelerated aging conditions used in this study (60°C, 85% relative humidity) are not typically encountered under natural field conditions, the aim of this approach is not to reproduce field conditions but to induce a standardized and highly repeatable decline in seed viability so that longevity parameters can be estimated. Previous work has indicated that p_50_ — the time required for seed viability, as measured by germination tests, to decline to 50% during storage — is one of the most robust and inter-study comparable metrics for assessing seed longevity (Hay et al., 2022). As the midpoint of the viability-loss curve, p_50_ reduces systematic differences arising from varying ageing temperature–humidity regimes, storage environments and laboratory conditions, and is therefore widely used in both accelerated ageing experiments and studies of natural seed longevity.

Meanwhile, the regression between L_50_ values (derived from accelerated ageing curve models for seeds collected in different years) and chronological seed age does not represent a direct physiological process. Instead, this regression was conducted to infer an upper bound of seed longevity for *S. rostratum* under laboratory conditions, given experimentally determined L_50_ values for seed cohorts of known ages. For this purpose, model selection should prioritize goodness-of-fit. We therefore compared polynomial regression with a Weibull-based model and found that all goodness-of-fit metrics consistently favored the polynomial regression (**Table 10**).

**Table 10:** Comparison of model fits for the relationship between seed age and L_50_ in *Solanum rostratum*.

Model	Equation	RSS	R^2^	AIC	BIC
Polynomial regression	y = 2.403749 -0.403234·x +0.016114·x^2	0.10	0.98	-23.97	-24.14
Weibull distribution	y = 15.040215·exp(-((x+23.463344)/17.811551)^{2.368872})	0.31	0.94	-13.81	-14.02

RSS denotes the residual sum of squares, and R² denotes the coefficient of determination. AIC and BIC are the Akaike and Bayesian information criteria, respectively, which compare model performance on the same dataset while penalizing model complexity (number of parameters). Except for R², lower RSS, AIC and BIC indicate a better fit.

Accordingly, our use of L_50_ as the target longevity parameter is not intended to represent an absolute field lifetime but rather to provide a standardized, comparable characteristic of viability decay that can reliably be used to contrast seed banks of invasive plants across conditions. Material available to this study was limited: the oldest accession dated from 2008 (15-year-old seed), followed by material from 2015 (8-year-old seed). In germination validation trials, the 2015 seeds exhibited a germination rate of 10.8%. Combining this empirical evidence with the polynomial regression provides an estimate indicating that, under dry storage at 4°C, the upper bound of *S. rostratum* seed longevity is approximately 8–9.91 years.

Because dry storage at 4°C generally offers more benign conditions than seeds experience in most natural habitats, this estimate should be interpreted as an upper bound for field longevity. Availability of additional seed lots collected between 2008 and 2015 would allow further refinement of this upper bound. Importantly, this method does not aim to determine the absolute lifespan of any single seed; rather, it quantifies the viability of an invasive species’ seed bank and its potential for persistence. By calculating L_50_ and comparing it with laboratory-derived reference values, one can infer corresponding seed ages and thereby inform targeted management strategies.

### 4.4 Limitations related to storage-time and cohort confounding

Previous studies have shown that increasing global CO_2_ concentrations and rising temperatures can reduce seed vigor without necessarily decreasing germination percentage (Hampton et al., 2013). Moreover, the maternal environment during seed development—particularly temperature—can markedly modulate seed dormancy, germination rate, and tolerance to deterioration in the subsequent generation (Penfield and MacGregor, 2016; Awan et al., 2018). Although all seed lots collected from 2008 to 2023 were stored at 4°C before testing, natural aging during storage cannot be completely separated from cohort-specific differences arising from environmental variation among collection years (e.g., drought versus wet years). This confounding effect represents a major limitation of the present study and may introduce uncertainty into the longevity estimates. Future work should therefore use seeds collected within a single year and test them after different storage durations, allowing storage-time effects to be more accurately distinguished from maternal environmental influences.

### 4.5 Management implications and methodological considerations

Previous studies have shown that co-occurring species in semi-arid grasslands typically form transient or short-term persistent seed banks. The dominant companion species of *S. rostratum* in our study area—*Leymus chinensis* and *Agropyron cristatum*—are annual grasses (Sun et al., 2023), and most native annual grasses are known to produce short-lived seed banks lasting only 1–2 years (Gasque and García-Fayos, 2003). In contrast, the estimated longevity of *S. rostratum* in our study (9.91 years) exceeds the commonly recognized threshold for long-term persistent seed banks (>5 years). This suggests that, relative to both native and co-occurring invasive species, *S. rostratum* may possess an unusually persistent soil seed bank. Such prolonged persistence could contribute to its frequent re-emergence in invaded habitats and increase the difficulty of long-term management.

Persistent seed banks are a major challenge for controlling annual invaders, as they enable reinvasion even after aboveground plants are removed. Estimating seed longevity and its rate of decline is therefore crucial for designing soil seed bank regulation strategies and for integrating long-term monitoring into weed management programs. In practical management, failure to recognize the high persistence of the *S. rostratum* seed bank may lead to premature relaxation of control efforts, resulting in recurrent emergence following apparent aboveground clearance (Buhler et al., 1997; Cordeau et al., 2022). Such underestimation of seed-bank persistence can compromise management effectiveness and increase long-term uncertainty and economic costs associated with repeated interventions.

Although the exact burial history of seeds recovered from grassland and abandoned farmland cannot be determined—including the time of entry into the soil seed bank, burial depth, or pre-burial environmental exposure—this study does not aim to estimate the absolute age of individual seeds *in situ*. Instead, our approach focuses on quantifying the relative vigor and persistence potential of local *S. rostratum* seed banks using accelerated aging assays combined with Weibull distribution modeling. In practical applications, the primary objective is to assess whether the seed bank at a given invaded site retains high persistence potential, rather than to reconstruct the precise aging trajectory of seeds in the field. Habitat conditions may modulate seed deterioration rates to some extent, but they serve mainly as auxiliary factors in the interpretation of seed-bank vigor. By comparing the L_50_ values derived from standardized aging treatments, managers can determine whether a site harbors a short-lived or potentially persistent seed bank and thus make informed decisions regarding eradication timing, monitoring frequency, and long-term management strategies. Nonetheless, we acknowledge that controlled burial experiments using seeds of known age and initial vigor would further strengthen the mechanistic understanding of habitat effects and provide a more rigorous validation of this approach. Accordingly, the accelerated aging–Weibull modeling approach is not intended to replace long-term field monitoring, but rather to serve as a rapid and standardized diagnostic tool for identifying high-risk sites at early stages of invasion and for supporting stage-specific and adaptive management decisions.

This study demonstrates that accelerated aging combined with Weibull modeling provides a rapid, standardized, and reproducible method to estimate seed longevity. The L_50_ metric serves as a reliable indicator of viability decline and allows for direct comparison among populations. Compared with conventional germination assays, this approach greatly reduces time requirements and enhances predictive capacity.

If *S. rostratum* seeds exhibit a potential upper-limit persistence of approximately 10 years, management strategies should be planned across a multi-year horizon rather than relying on short-term control. In practice, once the vigor level of the local seed bank is assessed using the standardized L_50_ approach, management intensity can be aligned with the inferred age or persistence potential of the seed bank. For example, if the estimated seed-bank vigor corresponds to that of 1–2-year-old seeds—indicating relatively high persistence potential—annual pre-flowering mowing or plant removal should be consistently implemented, and seed-bank vigor should be re-evaluated each year using the same accelerated-aging and Weibull-based method for estimating L_50_. A continuous suppression period of 4–5 years is recommended to ensure substantial depletion of the viable seed reservoir. When subsequent assessments indicate that the seed bank has transitioned to a lower-vigor, medium-longevity state, management frequency can be gradually reduced to lower economic costs while still maintaining effective suppression. By integrating standardized seed-bank vigor assessment with stage-appropriate management intensity, this approach provides an operational framework for designing ecologically sustainable and cost-effective control strategies for *S. rostratum*.

Nevertheless, accelerated aging is an artificial simulation that may not fully replicate natural decay. To improve model robustness, future research should incorporate long-term field storage experiments and expand sampling across additional populations and collection years.

## 5 Conclusion

This study demonstrates that the invasive annual *S. rostratum* exhibits consistent seed longevity across geographic origins, indicating that seed persistence is largely unaffected by environmental heterogeneity. Seed viability declines predictably with chronological age, and accelerated aging modeling provides reliable estimates of maximum seed lifespan, with projections suggesting persistence of up to a decade.

These findings highlight the ecological resilience of *S. rostratum* seed banks, underscoring their contribution to invasion persistence and management challenges. Importantly, the Weibull-based aging framework offers a practical and quantitative tool for assessing seed viability dynamics, which can inform predictive models of invasion risk and guide the design of seed bank depletion strategies. Beyond this species, the approach has broader applicability to other invasive annuals, providing a generalizable method to link seed traits with invasion ecology and management.

## Data Availability

The original contributions presented in the study are included in the article/Supplementary Material. Further inquiries can be directed to the corresponding authors.
